# Fecal elimination of fluralaner in different carnivore species after oral administration

**DOI:** 10.3389/fvets.2024.1279844

**Published:** 2024-08-16

**Authors:** Philippe Jacques Berny, Dalil Belhadj, Bernadette España, Alexis Lécu

**Affiliations:** ^1^UR-ICE Vetagro Sup, Lyon, France; ^2^Muséum National d'Histoire Naturelle, Paris, France

**Keywords:** antiparasitic drug, isoxazoline, fluralaner, carnivore, fecal elimination, external parasiticide

## Abstract

Fluralaner is a recent external parasiticide, first of a new class of drugs (isoxazoline parasiticides). It is widely used in companion animals both for its wide spectrum (fleas, ticks and other mites) but also for its ease of use (oral tablets given once for 1 to three months). It is known to be eliminated primarily via the feces (>90%) as the unchanged compound. In zoo carnivores, controlling external parasites is also important and there are no specific products with a marketing authorization to control them. The first objective of this study was to evaluate the pharmacokinetic profile of fluralaner in zoo carnivores. The second objective was to demonstrate that fluralaner can be eliminated over a prolonged period of time, thereby raising questions about its potential impact on non-target species such as arthropods. After adjusting the oral dose using allometric equations, animals were dosed and fecal samples were collected on a regular basis for up to three months to determine the fecal elimination curve of fluralaner as a surrogate of plasma kinetics (for ethical and safety reasons, plasma samples were not considered). All samples were analyzed with a validated LC-MSMS technique. Our results show that, despite limitations and a limited number of animals included, most carnivores eliminate fluralaner in their feces for several weeks to months (in Lions, fluralaner was still detectable after 89 days). To our knowledge, this is the first study demonstrating such a long elimination period in animals. Further studies would be required to investigate the risk associated with the presence of active residues in other carnivore feces for the environment, especially in dogs and cats, considering the large use of this class of compounds.

## Introduction

Few studies have documented external parasites that may be observed in captive wild animals in zoos. The Zoological Park Foundation of São Paulo (FPZSP) described and counted the total number of species of individual ticks observed in the zoo. Over 500 specimens were counted, belonging to 10 different species (1).

Controlling external parasites in captive animals in zoos is important, as external parasites may convey zoonotic diseases to personnel (caretakers, veterinarians…) and, to a lesser extent, to visitors of the park but also represent a major source of veterinary care for animals, as these parasites can have a strong negative impact on animal health [see for instance (2–4) for more information].

Unfortunately, as of today, there is no authorized external parasiticide for the prevention/treatment of parasitic diseases in wild, captive carnivores in zoos. As a consequence, veterinarians apply the “cascade” principle to select an appropriate drug to use as an external parasiticide in zoo animals (European directive 2001/82, replaced by RE 2019/6). A summary of the species of fleas and ticks identified in a selection of wild carnivores included in this study is presented in Supplementary material (1).

Fluralaner was the first isoxazoline approved for use in the EU. It is a non-competitive inhibitor of both the glutamate and GABA receptor-gated channel, with a higher selectivity for the receptor of insects and arachnids than mammals, thus providing a wide margin of safety in vertebrates (5). Fluralaner is a broad-spectrum external parasiticide (fleas, ticks, demodectic, sarcoptic and notoedric mange). Specific information regarding the pharmacokinetic profile of fluralaner in dogs and cats has been published (6, 7). After oral administration, the relative bioavailability is inversely related to the dose administered (20 to 34%). Presence of food greatly increases the oral absorption of isoxazolines (8). In dogs, bioavailability of fluralaner is doubled if the animals are fed prior to the administration of the drug (9). Fluralaner has a half-life of 11–13 days in dogs. Topical administration of fluralaner in dogs results in 22–25% bioavailability, with half-lives between 17 and 21 days (7). The pharmacokinetic profile of fluralaner shows a more stable plasma concentration after topical administration (plateau between 7 and 63 days). Fluralaner is highly protein-bound (>99%) and primarily eliminated via the bile (8). According to the CVMP (10) consulted online on June 1^st^, 2022, 90% of the administered dose is eliminated unchanged in the feces, resulting potentially in dissemination in the environment of fluralaner for 3 months as an active substance (see Environmental risk below). In cats, only topical administration data have been published and, overall, the pharmacokinetics of fluralaner in cats appears quite similar to PK in dogs. The half-life of fluralaner in cats is shorter (12–13 days) and the plateau is less prominent (7). In wildlife, a pharmacokinetic paper was also recently published (11) showing that the PK profile of fluralaner in black bears after administration of fluralaner orally at 25 mg.kg^−1^, showed a shorter half-life of 4.9 days.

Recently, an efficacy and safety trial was conducted in wombats (*Vombatus ursinus*), with pour-on preparations administered at either 25 or 85 mg.kg^−1^ (12). The elimination half-life was between 40 (25 mg.kg^−1^) and 166 days (85 mg.kg^−1^), much longer than in any other species. The authors suggested that, because of their lower metabolic rate than any other mammalian species, wombats were likely to display prolonged half-lives.

Available literature shows that fluralaner is regarded as generally safe to use in dogs and cats, and this was confirmed in a large double-blind study in the US. Only minor adverse reactions were detected in 221 dogs treated (6% displayed vomiting, 4% alopecia) (13). Because Isoxazolines are GABA antagonists, neurological disorders may be observed in vertebrates, especially dogs or cats and severely affected animals may need supportive care (14). These adverse reactions appear generally of good prognosis. In a recent investigation of severe adverse effects recorded with external parasiticides in dogs and cats (15), the overall incidence of severe adverse effects was 1/250,000 doses used and even less for lethal cases attributed to these treatments. Among these antiparasitic drugs, lethal cases related to the use of fluralaner in dogs were 5/1,000,000 doses, and even less in cats. These proportions make these adverse events very rare according to the current classification of adverse event frequency in the EU. A recent reappraisal (16) suggests that adverse events (as reported to the authorities) are more common than the Summary of Product Characteristics (SPC) would suggest but still quite rare, considering the major use of this class of compounds. In their efficacy and safety trial in wombats, Wilkinson et al. (12) showed that doses of 25 or 85 mg.kg^−1^ of fluralaner (pour on) were safe for use in these marsupials.

Some environmental data indicate that fluralaner may have detrimental properties on the environment. Specifically, fluralaner is considered highly toxic to aquatic invertebrates (*Daphnia magna*) with a No Observed Effect Concentration (NOEC) for reproduction of 0.047 μg.L^−1^ and to honey bees [VSDB, CVMP (10)]. Furthermore, it is quite stable in soil (DT_50_ up to 989 days in sandy loams) but poorly persistent in aquatic environments (DT_50_ around 3–4 days in aerobic waters), but quite stable in sediments (DT_50_ 112–195 days) and it has been classified as vP, T in the EU (10). Environmental Risk Assessment (ERA) was conducted according to the current guidelines and determined a Risk Quotient (RQ) <1 in all situations considered for laying hens. It should be reminded, however, that the recommended dosage in poultry is 0.5 mg.kg^−1^ twice 7 days apart, while most carnivore species receive between 25 and 56 mg.kg^−1^. Furthermore, this ERA has been conducted using some assumptions on the potential metabolism of Fluralaner and, as such, the guideline relies on fixed values not taking into account the biological (and statistical) uncertainty.

Because of their many advantages and also their safety profile in dogs and cats, isoxazolines appear as drugs of choice to treat wild carnivores present in zoos. A recent study in Raccoon dogs (17) showed that one oral dose of fluralaner was sufficient to cure these animals from sarcoptic mange, after 7 days. However, in the absence of any specific paper on the use of fluralaner in wild captive animals, determining the appropriate dosing-regimen in these species may not be feasible without any PK data.

The objective of this paper was, thus, to determine the pharmacokinetics of fluralaner in several carnivore species held in zoos and to compare it to the available data in dogs and cats. However, due to practical and ethical considerations, topical application as well as repeated blood sampling could not be considered and the study relied on the analysis of fecal samples collected on a regular basis after oral administration of fluralaner.

## Materials and methods

### Animals – dose determination – drug administration

Based on the species present at the Paris Zoo, the animals selected to be part of the study are described in Table 1.

**Table 1:** Characteristics of the animals involved in the study and allometric determination of the appropriate dose of fluralaner.

Species		Sex M+F = Total (treated)	Age (years) (min-max)	Husbandry	Food	Group	BW (kg)	P-Target (kcal^-1^.kg^-1^.j^-1^)	D-Target (mg)	Dose (mg.kg^-1^)	Tablets
*Panthera leo*	Felidae	2+2 = 4 (3)	6-13	Daytime outdoor Nighttime indoor	Eating 3 times a week	Males separated	175	3368	4109	23.S	2-3 (1400 mg)
*Puma concolor*	Felidae	1+3 = 4 (3)	1-12		Eating 3 times a week	Group daytime, isolated nighttime	63	1565	1909	30.3	1 (1400 mg)
*Panthera onca*	Felidae	1+1 = 2 (1)	6-8		Eating 3 times a week	Group daytime, isolated nighttime	81.2	1893	2309	28.4	2 (1400 mg)
*Cryptoprocta ferox*	Eupleridae	1+1 = 2 (1)	6-10		Eating 6 days out of 7	100% isolated	9.5	379	462	48.7	1 (250 mg)
*Suricatta suricatta*	Herspestidae	0+2 = 2 (1)	4-5	100% outdoor	Eating twice a day, 7/7 days	100% group	0.8	59	72	90.0	1 (112.5 mg)
*Canis lupus*	Canidae	1+1 = 2 (1)	10-11		Eating 3 times a week	100% group	34.9	1005	1226	35.l	1 (1000 mg)
*Speothos venaticus*	Canidae	1+2 = 3 (2)	2-5	Daytime outdoor Nighttime indoor	Eating 6 days out of 7	100% group	6	268	327	54.S	1 (250 mg)
*Nasua nasua*	Procyonidae	0+2 = 2 (1)	4-8	100 % outdoor	Eating twice a day, 7/7 days	100% group	4.8	225	274	57.2	1 (250 mg)
*Lutra lutra*	Mustelidae	1+1 = 2 (1)	7-9	Daytime outdoor Nighttime indoor	Eating twice a day, 7/7 days	Group daytime, isolated nighttime	6.8	295	360	52.9	1 (250 mg)

BW, body weight, P-target, energy consumption (target species), D-target, total dose of target animal/species, Dose, dose to be administered.

For all species, one individual was selected as a negative control animal. Age, sex, husbandry, food and housing conditions are described in Table 1.

The reference oral dose for dogs is reported as 40 mg/kg. According to the assessment report from CVMP (6), the minimum effective oral dose of BRAVECTO is 25 mg/kg [dose selection based on onset (within 8–12 h) and duration of activity (12 weeks)]. This translates into a dose range of 25 to 56 mg/kg when animals are dosed with the commercial tablets.

There is no published dose for the administration of fluralaner in wildlife species. In order to adapt the dosage to these species, allometric scaling was considered using the following formula (18):


$$P={\mathrm{aW}}^{b}.$$

Where P is the metabolic weight, W is the body weight of the animal, a is the metabolic constant corresponding to the energy consumption of a normal adult individual (kcal/d). This constant has been defined for many different taxa. For most mammals, a = 70. B is a constant close to 0.75.

According to Huang and Rivière (19), the correct dose can be determined according to the formula:


$$D-\mathrm{target}=D-{\mathrm{reference}}^{*}P-\mathrm{target}/P-\mathrm{reference}.$$

The reference dose was set at 40.5 mg/kg (6, 20), as the median dose administered with oral tablets in dogs. For a 20-kg dog the formula gives:


$$D-\mathrm{reference}=810\mathrm{mg}\;\mathrm{and}\;P-\mathrm{reference}=662\mathrm{kcal}.{\mathrm{kg}}^{-1}.d^{-1}.$$

$$\begin{array}{c} \mathrm{Thus},D-\mathrm{target}=c^{*}P-\mathrm{target where}\;c=D-\mathrm{reference}/P\\ -\mathrm{reference}=1.22\mathrm{mg}.{\mathrm{kcal}}^{-1}.\mathrm{kg}-1.d^{-1}. \end{array}$$

Table 1 presents the dosages determined for each species.

Each individual received 1–3 tablets of fluralaner (Bravecto®, formulations from 112.5 mg to 1,400 mg per tablet), based on the dose determined (Table 2) and the actual BW of the animal on day 0 (D0). Tablets were administered with the food. Tablets were not divided, according to the summary of product characteristics and the dose administered was adjusted as closely as possible to the theoretical dose calculated. The recommended bracket doses (25–56 mg/kg in a 20-kg dog) were used as a guideline (adjusted to the body weight of the animals) to ensure that the actual dose administered would remain in the acceptable margin. For instance, for the Iberian Wolf: using the dose range 25–56 mg/kg gave a D-reference of [500–1,120] mg, thus c = D-ref/P-ref = [0.76–1.69], i.e., D-target = [763–1,698] mg, resulting in a reference range of [21.9–48.6] mg.kg^−1^. The actual doses administered were between 28 and 30 mg.kg^−1^. This was checked for all species.

**Table 2:** Bravecto administration: actual dose (mg.kg^−1^ body weight) and extrapolated acceptable range based on the 25–56 mg.kg^−1^ BW dose in dogs and allometric extrapolation.

Species (n)	BW (kg) (median)	Tablets	Actual dose (mg.kg^−1^)	Extrapolated acceptable range^a^ (mg.kg^−1^)
Lion (3)	175	2–3 (1,400 mg)	[16.0–24.1]	14.6–32.5
Mountain lion (3)	63	1 (1,400 mg)	[20.8–25.0]	18.9–41.9
Jaguar (1)	81.2	2 (1,400 mg)	34.5	17.7–39.4
Fossa (1)	9.5	1 (250 mg)	26.3^b^	30.3–66.9
Iberian wolf (1)	34.9	1 (1,000 mg)	28.6	21.9–48.6
Bush dog (2)	6.0	1 (250 mg)	[40.3–43.1]	33.9–75.5
Suricate (1)	0.8	1 (112.5 mg)	125^b^	85.5–124.6
South American Coati (1)	4.8	1 (250 mg)	52.1	35.6–79.2
European otter (1)	6.8	1 (250 mg)	36.8	32.9–73.3

a Allometric extrapolation based on metabolic weight p = 70*(BW) 0.75.

b Slightly lower (Fossa) or higher (suricate) than extrapolated dose-range.

### Sample collection and storage

Feces were sampled on the floor, in priority on concrete ground in indoor facilities when animals were isolated. Fecal sampling was performed by keepers with gloves, transferred to sterile vials, then saved at −80°C until transfer to the lab.

Concerning animals living in groups days and night, markers were added to individual diets with items that can be visually detected in feces at sampling. Those markers were adapted to each species individual to maximize observance: e.g. watermelon seeds for *Nasua*, cooking food coloring liquid for Panthera, … Eventually, for some of them, an observer watched for the precise moment and location of defecation and stools were collected right after.

Each animal served as its own control (1–7 days prior administration) and negative control individuals were included in the study when at least 2 animals were present. The sampling schedule was based on the PK data available in dogs and cats (21). Only fecal samples were collected, for obvious reasons (limited manipulation of wild animals for ethical and safety reasons). If the « Zoo directive » (Directive 1999/22/EC) is promoting research in zoos, it should be within the respect of animal welfare. Therefore, this first study was meant to be non invasive by collecting fecal samples. At the time of this first study, blood could be collected either during chemical restraint, or on very few individuals, through medical training. Blood pharmacokinetic studies could be run in the future, but when the pool of trained animals is large enough.

Fecal samples were collected at days −7, +1, +7, +14, +21, +28, +35, +42, +49, +56, +70, +84, +98 and + 112 after treatment.

All samples were transferred to the lab and stored at −26°C until further analysis. During the development and validation of the method storage stability was determined (>1 month with no loss). All samples were shipped and analyzed on a weekly basis.

### Fluralaner analysis

Based on published techniques (21), it was decided to develop the techniques for all isoxazoline parasiticides.

Chemical reference substances of Fluralaner, Sarolaner, Afoxolaner and Lotilaner were purchased from CliniSciences (MCE Nanterre France, purity 99.95%). Acetonitrile of HPLC grade Ammonium hydrogen carbonate and ammonia for mobile phase were obtained from Merck Company (Darmstadt, Germany). Water was deionized and purified (Milli-Q system) (VEOLIA WATER TECHNOLOGIES, France) and was used to prepare all aqueous solutions. The Original 2003 QuEChERS method was used for the preparation of samples before analysis by LC–MS. The Extraction pouches contained 4 g MgSO4 and 1 g NaCl and were purchased from Interchim (Montluçon, France).

The apparatus was an LC–MS/MS system consisting of an Agilent 1,260 affinity II with a 100 μL sample loop and a 6470A triple quadrupole mass spectrometer (Agilent technologies, les Ulis, France) with an electrospray ionization source (ESI).

The ESI was operated in the negative mode with the following fixed settings: capillary voltage 3 kV; gas temp, 240°C at a gas flow of 10 L/min nitrogen, Sheath gas temp 400°C at a gas flow of 11 L/min nitrogen, nebulizer 35 psi. Dwell time was 50 ms for all compounds. The transition ions used for fluralaner were 554.0 (parent ion) and 534, 494, 424 for the product ions.

All standards and samples were analyzed on a VWR Lichrospher100 C_18e_ (100 mm × 4 mm ID, dp 5 μm) column. The mobile phase consisted of a mixture of acetonitrile and water (containing 10 mM of ammonium carbonate pH: 9 with ammonia) and was delivered at 0.5 mL/min using a gradient elution (30% water from time 0 to 0.2, 10% from time 0.2 to 5 min, and 30% from 5.5 min to 11 min, and total run time was 11 min).

**Figure 1:**
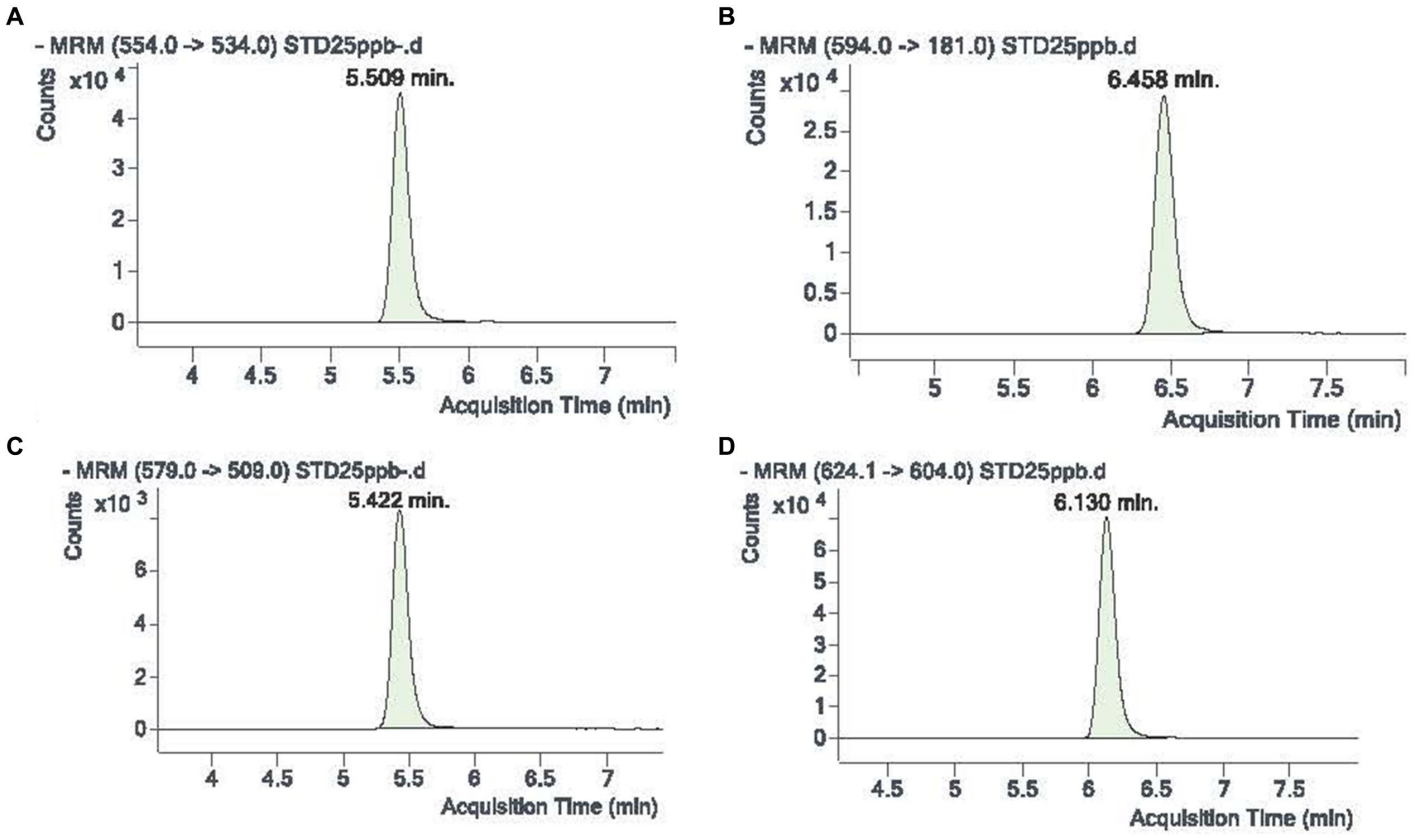
Retention time and transition ions for fluralaner **(A,C)** and lotilaner used as an internal standard **(B,D)**.

Stock solutions of parasiticides were prepared by dissolving the drug in acetonitrile at a concentration of 1 mg/mL then stored in 2 mL flask volumes at −20°C. Serial (working) dilutions of compounds were prepared at the concentrations of 2, 1, 0.5, 0.2, 0.1, 0.04 and 0.02 μg/mL. The final calibration curves ranged from 1 to 100 ng/mL. When a compound was used as the internal standard, it was diluted and added to the sample preparation so as to obtain a final concentration of 0.025 μg/mL. For PK studies, an internal standard was selected among the isoxazolines not administered to the animals. In the present study, lotilaner was used as an internal standard. Figure 1 shows typical retention time and transition ions for fluralaner (A, C) and lotilaner (B and D).

For feces analysis, well-homogenized samples were weighed and dried at 40°C overnight to eliminate water. 200 mg of the dried sample were weighed, 80 μL of IS solution (0.5 μg/mL solution), 0.52 mL of water, 200 mg QuEChERS salts and 1 mL of acetonitrile were added in a 5 mL plastic centrifuge tube. The sample was then mixed well by vortexing for 2 min and then centrifuged at 12,000 rpm for 5 min.

The upper clear layer solutions were transferred to auto-sampler vials and placed in the auto-sampler at 10°C for LC–MS/MS analysis. All results are reported in μg.kg^−1^ dry weight (dw).

Validation was carried out according to The International Conference on Harmonization (ICH) guidance Q2(R1) Validation of Analytical Procedures. Accuracy, precision and linearity of the calibration curve were determined. Intra-and inter-day precision were carried out on three different days. Each validation run consisted of a minimum of one set of calibration standards and six sets of QC samples at three concentrations. Stability of the stock solutions, auto sampler solutions, were determined.

Results of the validation procedure are shown in Supplementary Table S1.

In accordance with the results of the validation and the matrix effect, the samples were always being analyzed and quantified by reference to a range of supplemented samples carried out on the same day.

### Pharmacokinetic analysis

All pharmacokinetic data were computed using a non-linear model and “PK solver” in Microsoft-excel®. This approach has been used by Kilp et al. (21) and considered satisfactory by comparison with a standard PK software such as WinNonLIn®.

**Figure 2:**
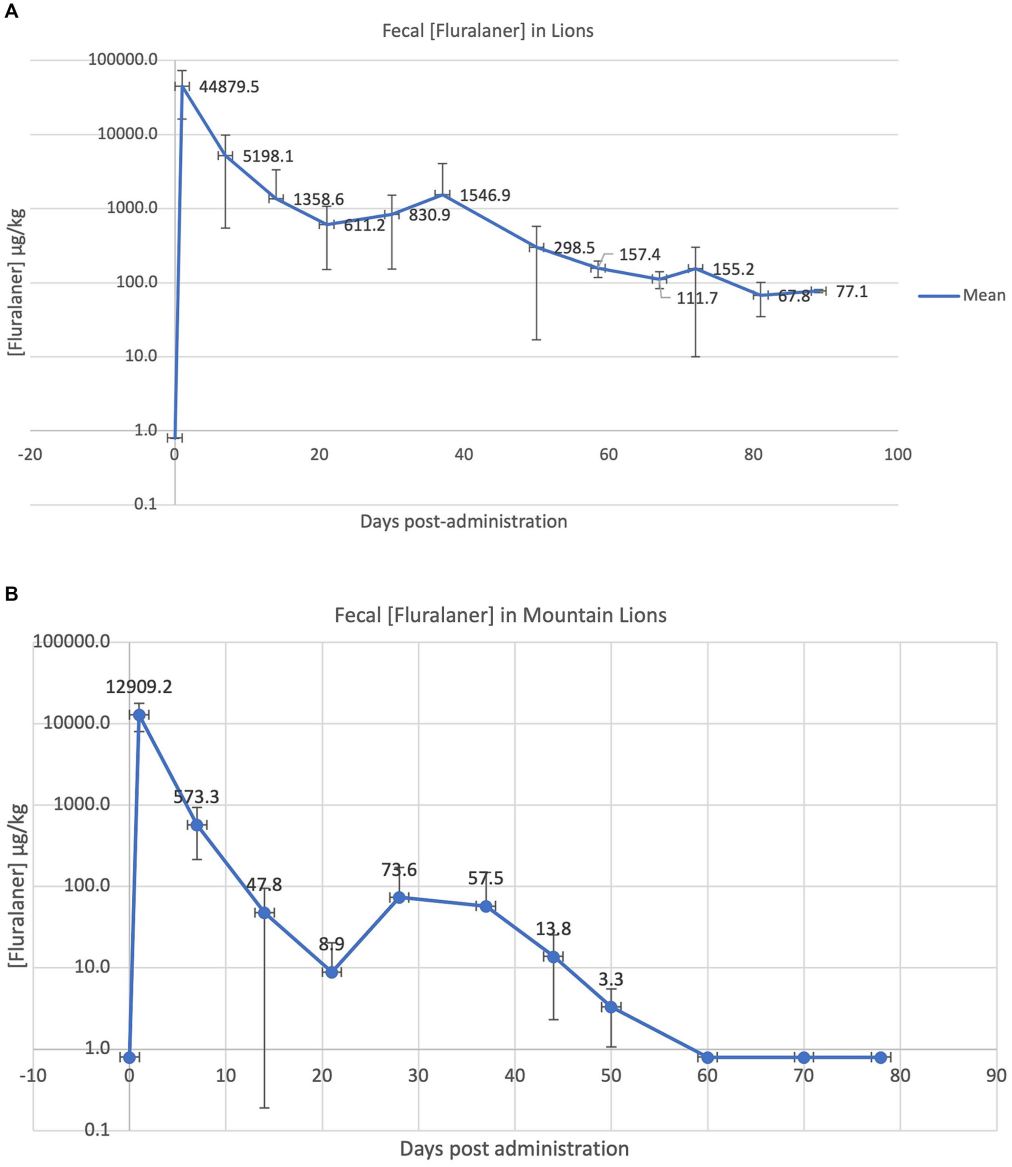
Fecal concentration of fluralaner in 3 Lions **(A)** or 3 Mountain Lions lions (μg.kg^−1^ dry weight) **(B)** during 3 months after an initial administration (Day 1). Vertical bars represent SD. Values < limit of detection (1.6 μg.kg-1) have been set arbitrarily at 1/2 the detection limit (0.8 μg.kg^-1^).

Only Lion and Mountain lion data (3 individuals+negative control for each species) are presented in Figure 2 as mean ± SD of the fecal concentration of fluralaner vs. time post administration. When data were LOD < Data<LOQ, an average value of 4 has been used (for graphical and PK analysis purposes). When concentrations were < LOD, ½ LOD value was used for the same purposes.

No statistical test could be carried out, except for a general comparison of the last day of detectable elimination in feces between canids and felids. This comparison was conducted with the r®software [after confirming the normality of the distribution (Shapiro’s test and QQplot and equality of variances)] by means of ANOVA. The significance level was set at 0.05. The relationship between Body weight and the duration of detection of residues (last day of detection) was determined using a linear model after log10 transformation of body weight for normality. For the validation process, linear regression and correlation coefficients were determined using the least square regression method (22) and a significance level of 0.05.

## Results

All animals could be treated and received a dose close to the theoretical estimated dose (Table 2), always within the acceptable range (except for Fossa: slightly below the lower limit). Tablets were well accepted and it was not necessary to consider a second administration. No adverse reaction was noted during the course of the study in any of the animals treated.

The mean fecal concentration of fluralaner in Lions and Mountain lions is displayed in Figure 2. Each point is the mean of 3 exposed animals, negative controls remained free of fluralaner residues over the entire course of the study (data not included). For graphical and mathematical purposes, values <Limit of detection were replaced with 0.8 (half the LOD).

PK parameters determined in all species are presented in Table 3 with means and SD when applicable (≥3 individuals).

**Table 3:** Pharmacokinetic parameters of the fecal elimination of fluralaner in canids and felids in captivity.

	Lion (*n* = 3)	Mountain lion (*n* = 3)	Fossa* (*n* = 1)	Jaguar (*n* = 1)	Suricate* (*n* = 1)
C_max_ (μg.kg-^1^ dry weight)**	44,880 ± 28,616	12,909 ± 4,954	370,295	16,089	15
t_max_ (d)**	1	1	1	7	14
AUC _(D0-D112)_ (d* μg.kg-^1^ dry weight)	232,796±95,614	54,158±19,755	1,373,277	145,337	105
AUC_(0-infini)_ (d* μg.kg-^1^ dry weight)	234,203±95,339	54,786±19,755	1,373,579	145,752	–
t_1/2_ (d)	12 ± 1	6 ± 1	3	8	–
MRT (d)	8 ± 1	3 ± 1	1	15	–
Last detection day (d)	89	49	35	72	14

	Iberian wolf (*n* = 1)	European otter(*n* = 1)	South American coati (*n* = 1)
C_max_ (μg.kg-^1^ dry weight)**	620	2,137	453
t_max_ (d)**	1	7	1
AUC _(D0-D112)_ (d* μg.kg-^1^ dry weight)	7,218	41,301	7,688
AUC_(0-infini)_ (d* μg.kg-^1^ dry weight)	7,260	41,354	8,998
t_1/2_ (d)	5	2	26
MRT (d)	6	16	38
Last detection day (d)	31	42	82

^*^Presence of residues in the control animal in the same cage. ^**^Note that, due to the sampling schedule, Cmax and tmax are only rough estimates of the actual kinetic parameters.

Some control animals living with their treated counterparts (Fossa and Coatis) were contaminated with fluralaner (ingestion of feces of treated individuals).

**Figure 3:**
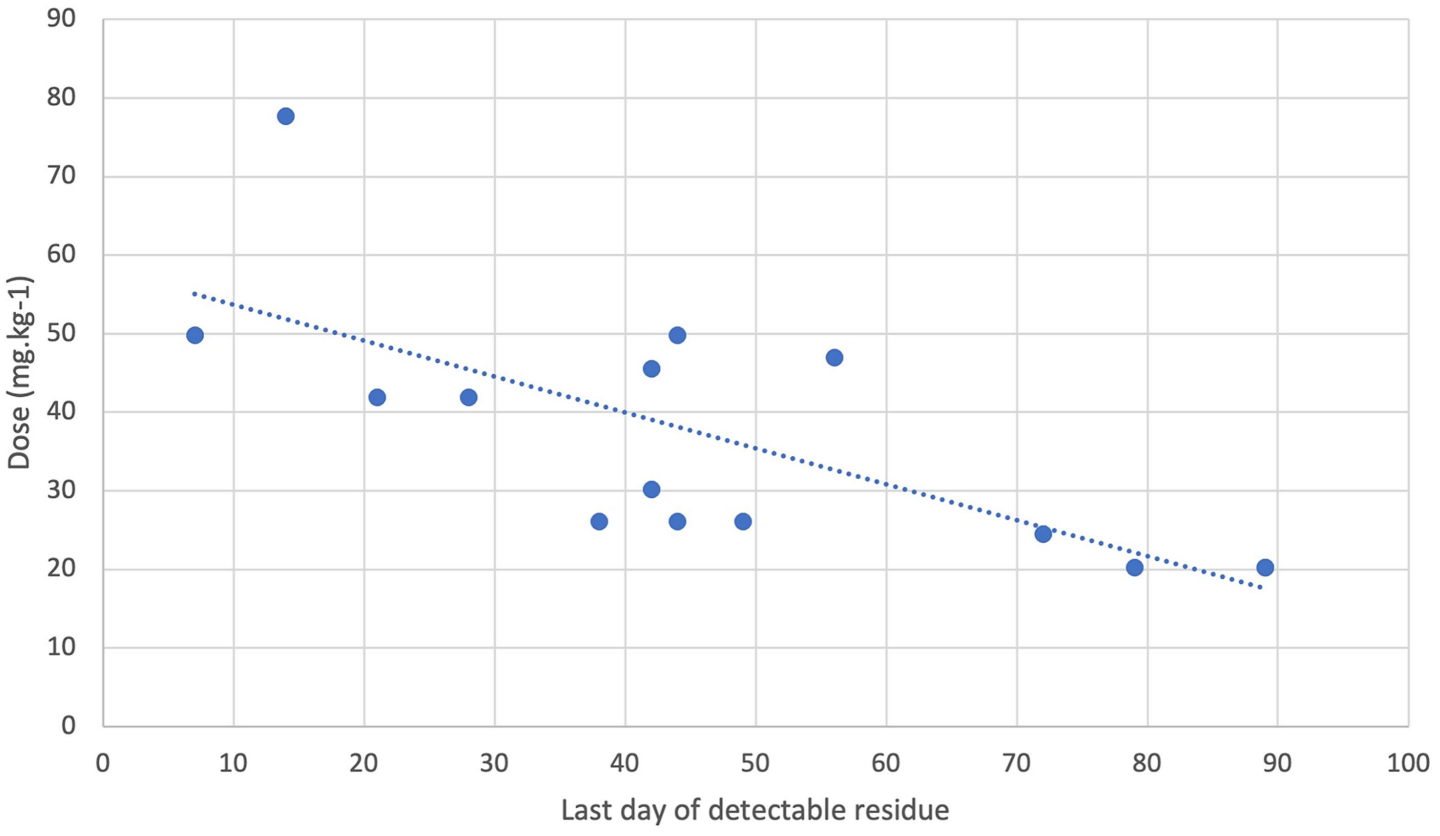
Relationship between dosage (in mg.kg^−1^) and last day of detection of residues in feces in carnivores.

Elimination half-lives can be extremely different. Overall, there was a negative log-linear relationship between the duration of elimination of Fluralaner in the feces and the body weight of the animals included (or the dosage in mg.kg^−1^) (Figure 3) (*R*^2^ = 0.62 *p* < 0.05).

## Discussion

There is no information on the bioavailability or PK of fluralaner in zoo-carnivores. The literature available suggests that fluralaner may be less absorbed in dogs, and more rapidly eliminated in cats. We did not observe such a difference between Felids and Canids, but the limited number of individuals in each species should be kept in mind before drawing definitive conclusions. Furthermore, there was a wide variability between individuals and between days.

In dogs and cats, fluralaner has a long, dose-dependent half-life resulting in a 12-week efficacy period (3). Half-lives are between 11 and 13 days in dogs after oral administration and appear to be longer in cats. Similarly, the elimination half-life is dose-dependent in wombats (12) ranging from 3 days at 25 mg.kg^−1^ to 37 days at 85 mg.kg^−1^. These values cover a wider range than those measured in dogs and cats. In a PK study in black bear cubs at 25 mg.kg^−1^, the PK profile of fluralaner appeared quite similar to our data, with an elimination half-life t_1/2_ of 4.9 days (11).

Unfortunately, there is no published data on the elimination profile of fluralaner in feces, but information available suggests that fluralaner is primarily eliminated as the parent compound in rats, dogs and chickens and that up to 90% of the administered dose is eliminated unchanged in the feces (6). There appears to be species-specific differences, rats excreting more rapidly and more intensively fluralaner than dogs.

Comparing fecal elimination and plasma PK profile is not a standard practice in PK, but it can be accepted in this experiment since (1) >90% of fluralaner is eliminated in the feces; (2) there is no metabolism and fluralaner is eliminated unchanged. Therefore, our results suggest that, despite wide variations between species, despite important intra and inter-day variation in excretion, the elimination half-life as measured by fecal concentration is a good surrogate to standard plasma elimination half-life, with a general trend towards longer elimination in larger animals, as was already observed between dogs and cats (7). The large range of body weight of the animals included in this study strengthens this suggested trend.

These results confirm the need for allometric scaling of the dose to be administered in order to avoid overdose or low dose in such a wide array of species, as suggested in the SPC and using the constant developed by Hainsworth (18). Appropriate dosing is important to provide rapid and effective control of external parasites in highly valuable animals. There is no evidence of development of resistant strains of external parasites so far, but some recent papers describe some degree of cross-resistance between fluralaner and permethrin in house flies (23), and it is a general recommendation to avoid low dosing in order to decrease the risk of potential selection of resistant strains of parasites.

Our data also suggest that the duration of action of fluralaner in captive wild carnivores may be quite variable across species. Most notably, lions have a much longer elimination half-life than all other species, while fluralaner appears to disappear rapidly from feces in smaller species (suricate, fossa, otter). It would be quite interesting to develop the monitoring of these animals for external parasite infestations to confirm the appropriate treatment duration, but also to increase the number of animals in each species (even to include other zoos in the experiment) to confirm our preliminary trial. We cannot exclude different biotransformation pathways in wild carnivores. Published information on the metabolism of isoxazoline ectoparasiticides is extremely limited. We could find evidence of P450 involvement in some insect species (24) therefore, apart from allometric difference across species, P450 metabolism may also play a role in the more rapid and intense elimination of fluralaner in some species like suricates. In order to extend the use of fluralaner, it could be advocated to investigate inter species metabolic differences using liver microsomes of several different species as suggested in a recent scientific opinion of EFSA (25).

Because we could not compare plasma and fecal kinetics, conducting a comparative study in several species would definitely strengthen our discussion on the use of fecal samples as surrogate samples for PK estimation in wild animals.

As one may expect working with captive wild animals, many limitations and biases can be described.

Among the limitations: the limited number of animals, the difficulty of collecting samples (thereby resulting in potential cross-contamination between individuals as observed with suricates) is also a problem for consistent monitoring. Most species were housed in groups and dyes were added to individual diets to help distinguish fecal samples, but degradation/dissipation of the dye, made it sometimes difficult to distinguish between individuals.

Fecal samples cannot be considered as homogenous and consistent biological samples. Both the nature and the amount available on each day could vary dramatically: from a few grams to several hundreds, fecal samples rich in hair or other digestive items. Careful steps were included by the team to collect the entire fecal output as often as possible and to homogenize the sample before analysis. Similarly, it was not always possible to collect all individual fecal samples on the same day, and analytical results were then averaged over a period of 2–3 days (after 3 weeks post administration).

No blood sample could be taken. Some animals are currently enrolled in operant conditioning for easier examination procedures, but this was not sufficient to ensure proper sample collection and safety of personnel (26).

It could also be argued that it is often recommended to conduct PK studies on fasted animals to reduce the variability linked to the feeds present in the digestive tract. It has been demonstrated (9), however, at least in dogs, that fluralaner absorption was enhanced and more consistent when the drug was administered slightly before or during the meal.

Our experimental design did not allow for proper and precise determination of C_max_ and T_max_, therefore these parameters should only be considered as indicative preliminary estimates.

Physico-chemical characteristics of fluralaner [see Veterinary Substances Data Base – VSDB (27) consulted on November 16, 2022] indicate that it is a lipophilic substance, with a DT_50_ of 60 days in soil [and much higher, close to 1,000 days according to the CVMP assessment report, CVMP (10)], which is not degraded by hydrolysis. The Summary of Product Characteristics (consulted on November 29th, 2022) states that « Fluralaner has been shown to be very persistent in soil under both, aerobic and anaerobic conditions. Fluralaner degrades in aquatic sediment under anaerobic conditions while it has been shown to be very persistent under aerobic conditions» and a specific assessment has been considered for the marketing authorization of EXZOLT® (used in poultry) (10). This regulatory assessment clearly states that Fluralaner is classified as vP, T in soil and aquatic aerobic sediments, but that it is not cumulative.

Fluralaner is a potent GABA and glutamate inhibitor. Its toxicity to insects or invertebrates has not been fully evaluated. Nevertheless, it is classified as highly toxic to *Daphnia magna* (aquatic invertebrate). The assessment report for EXZOLT® mentions an LC_50_ of 0.047 mg/L in this species and there is published evidence indicating that it is toxic to insect pests at low doses (28). It has been shown to be toxic to Lepidoptera insects as well (29).

Finally, it is important to stress that fluralaner is eliminated in feces as an active substance for several weeks (up to 3 months in our study). There is evidence that feces of carnivores can be degraded by dung beetles (30). Consequently, the long-term excretion as well as potential persistence of fluralaner residues in feces from carnivores could result in non-target poisoning of various insect species, including dung beetles and flies. Our data support the conclusions from a recent study showing that isoxazolines can be excreted in the environment and be found in hair but also in urine samples from treated dogs (31). Our results show, however, that the major route of environmental contamination could be via the feces, as the concentrations detected in fecal samples throughout the study are much higher than the measured values in urine or hair samples (up to 44,000 μg/kg in feces, vs. 1,000 μg/kg in hair and 1 μg/L in urine). As a consequence, fecal elimination is, by far, the most important route of environmental contamination (31).

## Conclusion

Because it is easy to use and administer, because it has an insecticidal effect on dog and cat fleas for 12 weeks, but also because it is a broad-spectrum antiparasitic agent, fluralaner is very commonly prescribed and used in companion animals and many animals are under permanent antiparasitic treatment. Our work suggests:

1. That the elimination kinetics may vary across species, suggesting the need for more data before extrapolating from one species to another;
2. That the repeated use of fluralaner in carnivores may result in prolonged elimination of a potent insecticide in the environment. Therefore, some management actions should be considered, to avoid environmental contamination, such as collection of stools by the owner for destruction (garbage/incineration elimination). Our data also support the need for some environmental risk assessment of veterinary drugs intended for use in companion animals, which is not currently considered in the EU, at least for drugs acting on species present in the environment, such as antibiotics and antiparasitic agents, as already suggested by Wells and Collins.

Future works on isoxazolines should certainly focus on dogs and cats and include all currently marketed compounds. More environmental toxicity data would be interesting as well to develop appropriate ERA scenarios. For instance, testing the toxicity of feces on dung beetles (as currently done for avermectins) house flies or other invertebrate species feeding on fecal material is necessary to determine environmental toxicity. These data could then be used to derive proper ERA scenarios and suggest adapted mitigation measures for these long-lasting parasiticides.

## Data availability statement

The original contributions presented in the study are included in the article/Supplementary material, further inquiries can be directed to the corresponding author.

## Ethics statement

The animal study was approved by VetAgro Sup, campus vétérinaire Ethics committee. The study was conducted in accordance with the local legislation and institutional requirements.

## Author contributions

PB: Supervision, Writing – original draft. DB: Investigation, Writing – original draft. BE: Investigation, Methodology, Writing – original draft. AL: Supervision, Writing – review & editing.
