# Supplementary material for: Fecal elimination of fluralaner in different carnivore species after oral administration
Source: Front Vet Sci. 2024 Aug 16;11:1279844. doi: 10.3389/fvets.2024.1279844 (PMC11362066; doi:10.3389/fvets.2024.1279844)
Supplement: Supplementary file 1 [file Table_1.docx]

Supplementary Table 1: Flea and tick species identified in wild carnivores (adapted from literature review by Belhadj, 2021) – *no species of fleas or ticks identified in Fossas

| Group | Species | Lion | Jaguar | Mountain lion | Suricate | Iberian wolf | Bush dog | South American coati | European otter |
| --- | --- | --- | --- | --- | --- | --- | --- | --- | --- |
| *Fleas* | *Ctenocephalides felis felis* |  |  |  |  |  |  | X |  |
|  | *Echidnophaga bradyta* |  |  |  | X |  |  |  |  |
|  | *Echydnophaga suricatta* |  |  |  | X |  |  |  |  |
|  | *Pulex irritans* |  |  |  |  | X |  |  |  |
|  | *Pulex simulans* |  | X | X |  |  |  |  |  |
|  | *Rhopalopsyllus lutzi lutzi* |  |  |  |  |  |  | X |  |
| *Ticks* | *Amblyomma brasiliense* |  |  |  |  |  |  | X |  |
|  | *Amblyomma cajennense* | X | X | X |  |  | X |  |  |
|  | *Amblyomma ovale* |  |  |  |  |  | X | X |  |
|  | *Amblyomma parvum* |  | X | X |  |  |  |  |  |
|  | *Amblyomma tigrinum* |  | X | X |  |  |  |  |  |
|  | *Amblyomma triste* |  | X | X |  |  |  |  |  |
|  | *Amblyomma sp* |  | X |  |  |  | X | X |  |
|  | *Ixodes canisuga* |  |  |  |  |  |  |  | X |
|  | *Ixodes hexagonus* |  |  |  |  |  |  |  | X |
|  | *Ixodes ricinus* |  |  |  |  |  |  |  | X |
|  | *Rhipicephalus microplus* |  | X |  |  |  |  |  |  |

Supplementary table 2 Validation results for isoxazoline external parasiticide analysis in fecal samples

| Criteria | Afoxolaner | | Sarolaner | | Lotilaner | | Fluralaner | |
| --- | --- | --- | --- | --- | --- | --- | --- | --- |
|  | Plasma | Feces | Plasma | Feces | Plasma | Feces | Plasma | Feces |
| Specificity | yes | yes | yes | yes | yes | yes | yes | yes |
| LOD ng/ml   LLOQ  ng/ml  ULOQ ng/ml | 0.5  2.5  500 | 1.6  8  1600 | 1.25  12.5  500 | 4  40  1600 | 0.5  2.5  500 | 1.6  8  1600 | 0.5  2.5  500 | 1.6  8  1600 |
| Carry over  1000ng/ml sample/ Blank sample | 1.44‰ | 0.33‰ | 1.37‰ | 3.88‰ | 4.86‰ | 5.37‰ | 1.80‰ | 1.28‰ |
| Linearity  Correlation | 0.0357x+0.1178  R^2^ : 0.993 | 0.0326x+0.0707  R^2^ : 0.998 | 0.0032x-0.0042  R^2^ : 0.999 | 0.0014x-0.0005  R^2^ : 0.9997 | 0.0428x+0.1056  R^2^ : 0.998 | 0.0366x+0.0677  R^2^ : 0.998 | 0.0409x+0.00362  R^2^ : 0.999 | 0.0439x+0.0949  R^2^ : 0.993 |
| Recovery  2ng/ml  25ng/ml  200ng/ml | 96.4%  88.3%  80.9% | 81.8%  75.8%  74.5% | 82.4%  72.2%  68.6% | 42.2%  37.7%  34.2% | 85.3%  83.8%  74.0% | 71.3%  64.1%  63.0% | 117.7%  121.2%  136.3% | 134.3%  130.7%  135.2% |
| Matrix effect Bias in % | -12.1 % | -19.7% | -24.0% | -65% | -18.0% | -32.0% | 28.1% | 32.9% |
| Precision  Intra day  Inter days | 5.5%  8.5% | 12.7%  14.7% | 12.4%  15.9% | 13.6%  15.6% | 6.0%  15.6% | 10.0%  14.2% | 5.1%  8.3% | 4.4%  8.5% |
| Dilution integrity  In matrix sample  In mobile phase | -4.3%  -15.8% | 12.2%  -5.3% | 16.1%  3.5% | 15.3%  -2.3% | -19.9%  -14.2% | 5.9%  14.7% | 4.4%  -18.8% | -11.4%  -15.0% |
| Stability (Bias %)  Sample in auto sampler 24h | 5.3% | -3.3% | 14.5% | -7.3% | 5.9% | -2.2% | -9.4% | -4.1% |
